# Convergent and Discriminant Validity of the Test of Passive Aggression in a Psychotherapy Outpatient Sample

**DOI:** 10.3389/fpsyg.2021.723413

**Published:** 2021-12-01

**Authors:** Christian Günter Schanz, Monika Equit, Sarah K. Schäfer, Tanja Michael

**Affiliations:** Division of Clinical Psychology and Psychotherapy, Department of Psychology, Saarland University, Saarbruecken, Germany

**Keywords:** self-directed aggression, passive aggression, test development, self-harm, aggressive behavior

## Abstract

**Background:** While most clinical aggression questionnaires focus on the assessment of active aggression, the recently developed Test of Passive Aggression (TPA) assesses both self-directed (TPA-SD) and other-directed passive aggression (TPA-OD). Reliability and factorial validity of the TPA have been demonstrated in a clinical sample, while previous evaluations of convergent and discriminant validity were limited to student samples. The current study aimed at addressing this gap by demonstrating convergent and discriminant validity of the TPA in an outpatient sample.

**Methods:** Eighty-two patients admitted to an outpatient psychotherapy unit at Saarland University, Germany, participated in the preregistered study with an assessment of self-reported passive aggression, impulsivity, anger expression, self-compassion, self-esteem, and auto-aggressive mindset. Analyses used regression models with robust maximum likelihood estimations.

**Results:** Self-directed passive aggression showed a significant association with self-compassion, auto-aggressive mindset, self-esteem, and internal anger expression supporting the convergent validity of TPA-SD. Results on discriminant validity of TPA-SD were heterogenous at the first sight, revealing small associations of self-directed passive aggression with anger control but medium associations with impulsivity. However, exploratory analysis showed that the medium association with impulsivity was driven by the non-behavioral impulsivity dimension “inattention” and that both behavioral impulsivity dimensions (“motor-impulsivity” and “unplanned behavior”) demonstrated only weak associations with TPA-SD. Validity of TPA-OD was not supported by the current study.

**Conclusion:** Our findings provide evidence for the validity of the TPA-SD to outpatient samples. Future studies will need to analyze construct validity based on a nomological network using larger and more diverse samples.

## Introduction

Aggressive behavior is defined as any behavior harming oneself or others in active or passive ways (Allen and Anderson, 2017). Active-aggression harms by engagement in active behavior (e.g., cutting oneself) and passive-aggression by omission (e.g., neglecting own needs; Buss, 1961). In comparison to the general population, the prevalence of both other-directed (e.g., violence: 2% vs. 10%; Swanson et al., 2015) and self-directed aggression (e.g., non-suicidal self-harm: 4% vs. 21%; Briere and Gil, 1998) is higher in individuals with mental disorders. Other-directed aggression shows its strongest associations with psychosis and substance abuse (Fazel et al., 2018), and self-directed aggression demonstrates its strongest relationship with depressive disorder as well as borderline personality disorder (Favril et al., 2020). However, both forms of aggression are related to a broad range of mental disorders, including anxiety disorders as well as trauma- and stressor-related disorders (Oram et al., 2014; Bentley et al., 2015). Although, aggressive behavior is more prevalent in inpatients, mild to moderate other-directed aggression and non-suicidal self-harm are also common in outpatients (de Klerk et al., 2011; Genovese et al., 2017). Currently, neither psychotherapeutic interventions for the reduction of other-directed nor self-directed aggression, demonstrate sufficient evidence (Rossouw and Fonagy, 2012; Stephens-Lewis et al., 2019). Thus, patients with mental disorders are a relevant population for research into aggressive behavior. However, previous research remained almost exclusively restricted to active aggression. The recently developed Test of Passive Aggression (TPA) is the first clinical assessment of self-directed (TPA-SD) and other-directed (TPA-OD) passive aggression (Schanz et al., 2021). Convergent as well as discriminant validity, and retest-reliability of the TPA have been demonstrated in a student sample. Additionally, the two-factor structure of the TPA was supported by exploratory structural equation modelling, using data of 307 patients with heterogeneous diagnoses in an inpatient setting. However, to date, support for its convergent and discriminant validity from a patient sample is missing. The current study aimed to address this gap by investigating convergent and discriminant validity of the TPA in an outpatient sample.

### Constructs for Evaluating the Validity of the TPA

Self-directed aggressive behavior is assumed to be closely related to self-conscious emotions (Laye-Gindhu and Schonert-Reichl, 2005) and self-criticism (Gilbert et al., 2010). In line with this assumption, Schanz et al. (2021) found an association between TPA-SD and an auto-aggressive mindset in a student sample, which requires a replication in an outpatient sample.

Impulsivity, a disposition for unreflected and spontaneous behavior, is a predictor for other-directed (Bresin, 2019) and self-directed (Hamza et al., 2015) active-aggressive behavior. By contrast, passive-aggression harms by omission and should thus occur independently from impulsivity (Buss, 1961; Parrott and Giancola, 2007). This assumption has also been supported by our previous study.

Anger can result in different behavioral responses: internal anger expression, external anger expression as well as anger control (Spielberger et al., 1988). Previous studies showed positive relationships between higher internal anger expression and self-directed aggression, higher external anger expression and increased other-directed aggression, as well as higher anger control (AC) and decreased aggressive behavior (Parrott and Giancola, 2004; Roberton et al., 2015; Kuzucu, 2016; Lievaart et al., 2016). With respect to internal anger expression and external anger expression, a replication of this pattern is expected for both TPA scales. However, passive aggression is assumed to be rather independent from anger control, for the same reason described above for impulsivity.

Self-esteem describes a stable and trait-like evaluation of one’s own worth (Kuster and Orth, 2013). Whereas previous results regarding the association between self-esteem and other-directed active aggression are heterogeneous (Ostrowsky, 2010), the results regarding the association between lower self-esteem and higher levels of self-directed active aggression are homogenous (Forrester et al., 2017). Therefore, a medium association of TPA-SD and self-esteem is expected.

Self-compassion is defined as a kind and mindful way to deal with oneself in challenging times (Neff, 2016). As such, self-compassion can be interpreted as the opposite of a self-directed aggressive attitude. This notion is supported by previous studies that demonstrated a strong negative association between self-directed aggressive behavior and self-compassion (Xavier et al., 2016; Jiang et al., 2017). Correspondingly, an at least moderate association between TPA-SD and self-compassion is expected.

Even though, for a final prove of construct validity a nomological network would be needed (Cronbach and Meehl, 1955), the current study will provide first insights into convergent and discriminant validity of the TPA. To this end, the sum of the presence of expected correlations (i.e., at least medium associations of TPA-SD with an auto-aggressive mindset, internal anger expression, self-esteem, and self-compassion as well as at least medium associations of TPA-OD with external anger expression) and the absence of unexpected relationships (i.e., at most small associations of both TPA scales with anger control and impulsivity) will provide support for the TPA’s validity and form a foundation for its further investigation.

## Materials and Methods

### Participants and Procedure

Patients were recruited subsequent to their initial consultation at the Center for Behavior Therapy of the Saarland University and the Institute for Postgraduate Studies in Psychotherapy Saarbruecken. All patients were adults (age≥18years) and gave written informed consent according to latest revision of the Declaration of Helsinki. Eighty-two patients [62.20% female, *M*_age_=39.84 (*SD*=13.34)] were enrolled in the study. Diagnoses were based on the Structured Clinical Interview for Mental Disorders for DSM-5 – Clinical Version (SCID-5-CV; Beesdo-Baum et al., 2019). Affective disorders (44.87%) were the most prevalent main diagnosis, followed by anxiety disorders (25.64%) and trauma- and stress-related disorders (17.95%) with 39% of the patients being diagnosed with at least two mental disorders. The study was preregistered in the German Clinical Trial Register (www.drks.de, DRKS00017321).

### Measurements

Passive aggression was measured using the TPA. The TPA comprises two scales assessing self-directed (TPA-SD) and other-directed passive aggression (TPA-OD), with 12 items each. For English translations of all TPA items, see Schanz et al. (2021). TPA-SD exhibited good (*α*=0.82) and TPA-OD acceptable (*α*=0.78) internal consistency in the current sample.

Impulsivity was assessed with the 15-item short-form of the Barratt Impulsiveness Scale (BIS-15; Spinella, 2007). The BIS-15 comprises three subscales (inattention, motor impulsivity, and unplanned behavior). The global score and all subscales showed good internal consistency (*α*=0.80–0.85) in the present study.

Behavioral responses to anger were assessed using the Stait-Trait-Anger-Expression-Inventory-2 (STAXI-2). The STAXI-2 assesses internal anger expression (AX-I), external anger expression (AX-O) as well as AC using 26 items. An initially assumed differentiation in internal and external AC was not empirically supported in previous studies (Rohrmann, 2013). In the present sample, all STAXI-2 scales demonstrated acceptable to good internal consistency (*α*=0.77–0.84).

The auto-aggression scale of the Short Questionnaire for Assessing Factors of Aggression (K-FAF; Heubrock and Petermann, 2008) was used for assessing an auto-aggressive mindset (i.e., self-conscious emotions and self-criticism). It consists of nine items and demonstrated good internal consistency (*α*=0.81) in the current sample.

Self-compassion was assessed using the 24-item Self-Compassion Scale (SCS; Neff, 2016; Coroiu et al., 2018). In the current sample, the score of the SCS demonstrated good internal consistency (*α*=0.90).

Self-esteem was assessed with the Multidimensional Self-Esteem Scale (MSES; Schütz and Sellin, 2006), a German adaptation of the Multidimensional Self Concept Scale (Rotatori, 1994), comprising 32 items. In the present study, the MSES showed high internal consistency (*α*=0.92).

### Data Analyses

All analyses were performed using the *lavaan* package (Rosseel, 2012) in *RStudio* (RStudio Team, 2019). For the evaluation of convergent and discriminant validity, bivariate associations between passive aggressive behavior and all relevant measures were analyzed using regression analyses with robust maximum likelihood estimation. Additionally, on an exploratory basis, we performed multiple regression analyses with robust maximum likelihood estimation to analyze unique association between passive aggressive behavior and each study variable under mutual control for other study variables, age and gender. Effect sizes were interpreted following the guidelines proposed by Cohen (2013); *β*≥0.10: small; *β*≥0.30: medium; and *β*≥0.50: strong. A *post hoc* confirmatory factor analysis was conducted using the *lavaan* package, evaluating the two-factor structure of the TPA. Due to multivariate non-normality, the analysis was performed using diagonally weighted least squares (DWLS) estimation procedure and robust error estimators (Mîndrilã, 2010). Results of this analysis should interprete with caution as the current sample was not recruited in order to examine the factorial structure of the TPA (Worthington and Whittaker, 2006). However, as the TPA constitutes a newly developed instrument, we decided to include the analysis to provide the reader with all available information on TPA’s psychometric properties.

## Results

Results of factor analysis are presented in Table 1. Whereas, Comparative Fit Index (CFI), Tucker-Lewis Index (TLI), Root Mean Square Error of Approximation (RMSEA), and model test [*Χ*^2^(251)=278.44; *p*=0.113] supported the two-factor structure of the TPA, Standardized Root Mean Square Residual (SRMR) did not.^1^ Associations between the passive-aggression subscales and study variables are presented in Table 2. With respect to TPA-SD, all associations were in line with our hypotheses, except for the association with global impulsivity. An exploratory analysis of the subscales of the BIS-15 revealed a weak association between TPA-SD and unplanned behavior (*β*=0.22; *p*=0.047) as well as with motor impulsivity (*β*=0.19; *p*=0.088), but a medium-sized relationship between TPA-SD and inattention (*β*=0.45; *p*<0.001). In line with our hypotheses, other-directed passive aggression did not account for a significant amount of variance in impulsivity and AC. However, in contrast to our assumptions, the association between other-directed passive aggression and external anger expression (AX-O) was only small. Controlling the associations of TPA-SD with study variables for TPA-OD and vice versa, did not result in a relevant change of results (see Table 2). Moreover, results also remained stable when controlling for age and gender. In a multiple regression analysis including all study variables, only an auto-aggressive mindset (K-FAF) shared a unique amount of variance with self-directed passive aggression (TPA-SD), *β*=0.36, *t*(73)=2.67; *p*=0.009. For TPA-OD, in a multiple regression analysis including all study variables, only self-compassion (SCS) accounted for a unique amount of variance, *β*=−0.31, *t*(73)=−2.18; *p*=0.033.

**Table 1:** Results of confirmatory factor analysis.

*CFI*	*TLI*	*RMSEA*	*SRMR*	*Chi^2^*	*df*	*p*
0.97	0.97	0.04	0.12	278.44	251	0.113

CFI, comparative fit index; TLI, Tucker-Lewis index; RMSEA, root mean square error of approximation; and SRMR, standardized root mean square residual.

**Table 2:** Descriptive statistics of study variables and associations with Test of Passive Aggression (TPA) scales.

	Mean (*SD*)	TPA-SD				TPA-OD			
		*β* zero order	*β* controlled for age and gender	*β* controlled for TPA-OD	*β* controlled for age, gender and TPA-OD	*β* zero order	*β* controlled for age and gender	*β* controlled for TPA-SD	*β* controlled for age, gender and TPA-SD
AX-O	12.18 (3.83)	0.20	0.14	0.15	0.11	0.23	0.22	0.19	0.20
AX-I	20.26 (4.26)	0.40^**^	0.37^**^	0.39^**^	0.41^**^	0.14	0.14	0.05	0.05
AC	28.95 (5.45)	0.00	0.06	0.04	0.11	−0.14	−0.14	−0.14	−0.17
SCS	67.18 (18.30)	−0.44^**^	−0.37^**^	−0.39^**^	−0.35^**^	−0.30^*^	−0.31^*^	−0.20	−0.22
K-FAF	20.35 (8.24)	0.60^**^	0.54^**^	0.61^**^	0.54^**^	0.13	0.15	0.03	0.00
BIS-15	33.74 (8.02)	0.36^*^	0.29^*^	0.34^*^	0.30^*^	0.16	0.13	0.08	0.05
MSES	127.60 (32.32)	−0.50^**^	−0.47^**^	−0.51^**^	−0.52^**^	−0.06	−0.09	0.06	−0.03

*β*, standardized regression weights; TPA-SD, test of passive aggression – self-directed; TPA-OD, test of passive aggression – other-directed; AX-O, external anger expression; AX-I, internal anger expression; AC, anger control; SCS, self-compassion scale; K-FAF, short questionnaire for assessing factors of aggression; BIS-15, barratt impulsiveness scale-short form; and MSES, multidimensional self-esteem scale.

* Indicates *p*<0.05.

** Indicates *p*<0.001.

## Discussion

Self-directed passive aggression harms oneself by omission of need satisfaction and disengagement in positive experiences in consequence of negative self-evaluations (Schanz et al., 2021). Consequently, we assumed the TPA-SD scale to be negatively associated with measures of positive self-evaluation (i.e., self-esteem) and with measures assessing a kind and mindful way to deal with oneself (i.e., self-compassion) and to be associated positively with measures of negative self-evaluation (i.e., auto-aggressive mindset). Additionally, we expected the TPA-SD scale to be positively associated with other measures of self-directed aggression (i.e., internal anger expression, AX-I). These hypotheses were confirmed by the current study supporting the convergent validity of the TPA-SD scale.

With respect to discriminant validity of the TPA-SD scale, results were heterogeneous. In line with our hypotheses, the relationship between TPA-SD and anger control were small and non-significant. However, TPA-SD and impulsivity – measured using the global score of the BIS-15 – showed a medium-sized association. Further exploratory analyses revealed only weak associations between TPA-SD and the behavior associated subscales of the BIS-15 (i.e., unplanned behavior and motor-impulsiveness). By contrast, the in attention subscale of the BIS-15 measures a lack of focus and does not assess any behavioral engagement at all (Meule et al., 2011). Therefore, the medium-sized association between TPA-SD and BIS-15 global score does not contradict the discriminant validity of the TPA-SD. This notion needs to be considered when investigating the validity of the TPA based on a nomological network, which should include heterogeneous relationships between self-directed passive aggression and dimensions of impulsivity.

Individuals with passive-aggressive personality try to harm others by omission instead of active engagement (Parrott and Giancola, 2007). Thus, we assumed other-directed passive aggression to be moderately associated with measures of external anger-expression, but not with measures of impulsivity or impulse control. While the evidence for the latter hypothesis was convincing, the association between other-directed passive aggression and external anger-expression was only weak. Thereby, contradicting previous results that demonstrated a medium to strong association between TPA-OD and active aggression measured with the K-FAF (Schanz et al., 2021). However, it remains unclear if these diverging results may be accounted for by methodological differences, differences in sample characteristics, or differences in assessed aggression types. Thus, future studies need to assess a broad range of operationalizations of aggression in different samples to further explore the underlying latent constructs.

The current study was conducted for evaluating convergent and discriminant validity of the TPA by examining bivariate associations with related constructs. Sample size was limited by data collection in a clinical outpatient sample but followed recommendations for the development of psychometric scales (Hobart et al., 2012; Streiner and Kottner, 2014). However, this approach may neglect the robustness of single coefficient estimations. Simulation studies suggest that such estimations require larger sample sizes, i.e., *N*≥250 (Schönbrodt and Perugini, 2013). Therefore, our findings need to be replicated to provide more robust estimates of regression and correlation coefficients. Moreover, such studies would also allow for the use of more advanced methods (e.g., structural equation modeling) to investigate construct validity. Therefore, future studies exploring the psychometric properties of the TPA in clinical samples of larger size are needed.

In sum, this study enlarges the knowledge on psychometric properties of the TPA-SD by providing evidence for validity in an outpatient sample. The validity of TPA-OD was not supported by the current study. Building on the findings of the present study, future studies in large-scale clinical samples should further investigate the validity of the TPA-SD as the first clinical assessment of self-directed passive aggression to provide a comprehensive psychometric evaluation. These studies should particularly focus on the relationship between self-directed passive aggression and different dimensions of impulsivity differing in behavioral consequences.

## Data Availability Statement

The raw data supporting the conclusions of this article will be made available by the authors, without undue reservation.

## Ethics Statement

The studies involving human participants were reviewed and approved by Ethics Committee of Saarland University. The participants provided their written informed consent to participate in this study.

## Author Contributions

CS developed the study design, recruited the participants, analyzed the data, and prepared the materials and first draft of the manuscript. ME, SS, and TM participated in the development of the study design and reviewed the materials and manuscript. All authors contributed to the article and approved the submitted version.

## Conflict of Interest

The authors declare that the research was conducted in the absence of any commercial or financial relationships that could be construed as a potential conflict of interest.

## Publisher’s Note

All claims expressed in this article are solely those of the authors and do not necessarily represent those of their affiliated organizations, or those of the publisher, the editors and the reviewers. Any product that may be evaluated in this article, or claim that may be made by its manufacturer, is not guaranteed or endorsed by the publisher.

## Footnotes

^1^As DWLS estimations that were used due to the multivariate non-normality of the data assume data to be ordinally scaled, SRMR may underestimate the model fit (Mîndrilã, 2010).
